# Assessing Spatial Accessibility to Hierarchical Urban Parks by Multi-Types of Travel Distance in Shenzhen, China

**DOI:** 10.3390/ijerph16061038

**Published:** 2019-03-22

**Authors:** Langjiao Li, Qingyun Du, Fu Ren, Xiangyuan Ma

**Affiliations:** 1 School of Resource and Environmental Sciences, Wuhan University, Wuhan 430079, China; lilangjiao299@whu.edu.cn (L.L.); renfu@whu.edu.cn (F.R.); maxiangyuan@whu.edu.cn (X.M.); 2 Key Laboratory of Geographic Information Systems, Ministry of Education, Wuhan University, Wuhan 430079, China; 3 Key Laboratory of Digital Mapping and Land Information Application Engineering, National Administration of Surveying, Mapping and Geoinformation, Wuhan University, Wuhan 430079, China; 4 Collaborative Innovation Center of Geospatial Technology, Wuhan University, Wuhan 430079, China

**Keywords:** urban green spaces, accessibility, spatial disparity, 2SFCA, Shenzhen

## Abstract

Urban green spaces play a critical role in public health and human wellbeing for urban residents. Due to the uneven spatial distribution of urban green spaces in most of cities, the issue of the disparity between supply and demand has aroused public concern. In a case of Shenzhen, a modified Gaussian-based two-step floating catchment area (2SFCA) method is adopted to evaluate the disparity between park provision and the demanders in terms of accessibility at hierarchical levels under four types of distance (e.g., Euclidean distance, walking distance, bicycling distance, and driving distance), which is well aligned with hierarchical systems in urban green spaces in urban planning practice. By contrast and correlation analysis, among the four types of distance, the statistical correlations are relatively high between Euclidean distance and the other three. Nonetheless, the pattern of spatial accessibility under different type of travel distance is apparently variant. Accessibility calculated by Euclidean distance is overestimated relative to that of the other three, while the pattern of walking distance and bicycling distance is similar to each other. The choice of type of distance is worthy of caution when evaluating spatial accessibility by 2SFCA method. Results show that the accessibility to parks at all hierarchical levels is high particularly, particularly at the natural level. However, the disparity between the supply and demand is significant. The percentage of communities that have high population density but low park accessibility is over 40% (equivalent to approximately 55% of the population). The finding may provide implications on access to urban greens paces for urban planners and authorities to develop effective planning strategies.

## 1. Introduction

Urban green spaces, such as parks, forests, greenbelts, and residential greenery, play a critical role in the connection between human beings and the natural environment in urbanized areas, which provide physical, psychological, environmental, economic, and social benefits for city dwellers [1,2,3,4]. Urban green spaces covered with vegetation can contribute to improving air quality [5], alleviating urban heat island effects [6,7], and offsetting carbon emission [8,9]. Urban green spaces, especially parks, are ideal places for residents to relax, enjoy leisure activities, perform physical activity, and communicate. A large body of literature explored the associations between green spaces and health (for example, [10,11,12,13]). Greener living environments enhance human health, including reducing risk of chronic diseases (i.e., obesity, cardio-metabolic issues, and sedentary behavior) [13,14], alleviating stress-related psychosocial symptoms [15,16], improving self-esteem and mood [17], and facilitating social interaction [18,19]. Generally, the spatial distribution of urban green spaces is not homogeneous within cities [20,21,22]. It has stimulated concern among researchers and urban planners about social equity and environmental justice regarding urban green spaces [23,24,25,26].

Access to green spaces as components of environmental justice has got much attention from scholars and urban planners. Many studies have examined the disparity between the provision of urban green spaces and demand populations from the perspective of socio-economic status and demographics around the world [23,27,28,29,30]. For example, Boone et al. found that the whites are provided with more park acreage within walking distance than the blacks in Baltimore, Maryland [23]. In contrast, Xiao et al. has stated that the vulnerable groups were not disadvantaged over the affluent in Shanghai in terms of access to urban parks [28]. However, the results from empirical studies are far from reaching a consensus [31]. In Macintyre’s review, it showed that low-income communities of color in some instances have better access to health-promoting facilities than other groups, including parks [32], while a review on access to green space by [33] found that low-income communities of color experience lower park service than the white and affluent groups do. The measurement of access to parks may have a crucial effect on their findings [31,33,34]. How to model the access to urban parks is still a hot controversial topic in the study of urban planning and sustainability [21,27,35,36,37].

A growing body of literature has developed various indices and methods for assessing access to urban green spaces [27,34,35,38]. Nevertheless, the green spaces classification systems in urban planning practice were ignored. Urban planners and authorities categorized green spaces based on the characteristics of size, function, and amenities [39]. Green spaces on different functional scales cannot be substituted for each other. In evaluating access to urban green spaces, “functional levels” ranging from the street to city level should be involved [40]. In addition, the available green space services may vary due to the mode of transportation people choose to reach those spaces [41]. The manner in which people visit urban parks is uncertain. In summary, to our knowledge, studies on access to urban green spaces under multiple-types of distance has not been fully explored [35].

According to Penchansky and Thomas’s theory of access in 1981, the concept of access has been widely accepted as a multidimensional notion and depicted in terms of availability, accessibility, affordability, acceptability, and accommodation [42,43,44,45]. Several scholars have argued that the notion can be categorized into spatial and aspatial dimensions, or potential and actual dimensions [44,46,47]. Spatial access highlights the separation between supply (i.e., urban green spaces) and demand (i.e., population) and the way they connect in space [46], while aspatial access emphasizes nongeographic barriers including demographic, socioeconomic, cultural, and management variables [45,46,47]. One of the most common approaches to evaluating access to public and private facilities like health care, recreational amenities, food outlets, and parks is by measuring spatial accessibility.

Various factors influence spatial accessibility to green spaces, such as the provision of green spaces (i.e., locations, park numbers, size), the distribution of demand population (e.g., population size, population composition, neighborhood attractiveness), the barriers between green spaces and the demand population, and the metrics of access [24,48,49,50].

With respect to access measurement, diverse approaches, indicators, indices, metrics, and standards have been proposed to assess accessibility to urban green spaces. According to the review of Rigonlon [31], the measure of spatial access to parks can be categorized into three general approaches: (1) the spatial proximity approach, which measures the travel cost of the demanded population to the closest park, regardless of the park’s size and amenities; (2) the container approach, which measures the number or size of parks within a geographic unit or a given distance of a geographic unit; and (3) the coverage approach, which measures the ratio of demand to supply in a park’s supposed service area by, for example, simple buffer analysis, the network constrained service areas, the Thiessen polygon method, kernel density estimation, and gravity-based models. In particular, the gravity-based models have been one of the most popular methods for measuring accessibility [4,27]. The basic gravity models, based on Newton’s Law of gravitation, defined the accessibility as “the potential of opportunities for interaction” [51], which are conceptually more advanced in considering the distance decay effect. However, it is difficult to select a fit decay function and an appropriate impedance parameter β in the model [44]. Thus, an improved gravity model, the two-step floating catchment area method (2SFCA), first proposed by [52] and modified by [47], was introduced.

In the 2SFCA method, the distance decay effect was defined via a dichotomous function, which omits the distance decay within the catchment. Several scholars have attempted to compensate for the shortcomings of this method by integrating different distance impedance functions into the initial 2SFCA method, such as a kernel density (KD) function [44], discrete time zones with constant weights in each zone [53,54], a constant and gradual mixed decay function [55], a Gaussian function [27], or a logistic cumulative distribution function [56]. However, in all of these modified 2SFCA methods, a fixed catchment size was set for all facilities regardless of the characteristics of the facilities (e.g., size, level of service in urban planning). By avoiding the fixed catchment size for all supply and demand sites in 2SFCA, a few studies have improved upon the original 2SFCA based on a variable or dynamic catchment size on either the supply or population side [41,57,58,59]. Dony et al. divided park catchment size into five levels based on the attractiveness, which was defined as a function of park size and the number of amenities [41]. While this approach may make a conceptual improvement in assessing access to parks, determining an optimal attractiveness function is much more complicated and less intuitive than park size.

Moreover, the original 2SFCA method was based on the inappropriate assumption that all population travel to service sites by a single transportation mode [60]. In reality, the mode of transportation may vary among individuals and may be associate with the location of destinations. Thus far, only a few scholars have taken multiple transport modes into account on access to urban green spaces [35,38,41]. Dony et al. developed a variable-width floating catchment area (VFCA) method to assess accessibility to parks in Mecklenburg County, North Carolina and compared accessibility under four modes of transportation (e.g., bicycling, public transit, and walking) [41].

Although access to urban green spaces has been examined in many studies, access to green spaces in China’s megacities is little known [28,38]. In China, urban parks have been given priority in the metropolises to improve the urban environment [61]. An empirical study by Xu et al. estimated park accessibility by seven indicators derived from travel time under four transport modes, and stated that social inequalities in park accessibility by walking and public transit were significant in Shenzhen [38]. However, the indicators of park accessibility neglected the distribution of the demanded population. The implications for urban planners in figuring out the mismatch between the provision of urban green spaces and demanding people may be limited.

As previously stated, our study attempts to introduce a modified Gaussian-2SFCA method to evaluate spatial access to parks in Shenzhen, China. Taking the levels of parks and multiple transportation networks into consideration, the paper aims to answer the following two questions: (1) Do the metrics of distance under multiple modes of transportation affect park accessibility; (2) Does the disparity between park provisions and the demanded population exist? Where are the communities that have a shortage of green services in Shenzhen? More importantly, our analytical study contributes to enriching the relevant theoretical foundation regarding park accessibility modelling and provides some insights into green urban planning for local administrators and planners.

The remainder of this paper is structured as follows: Section 2 makes a brief introduction to the city of Shenzhen and its park classification system. Section 3 depicts the modified Gaussian-based 2SFCA model, as well as the setting of parameters in detail. In Section 4, the correlations and variations among four types of distance for measuring accessibility are systematically explained. Conclusions, limitations, and future work are presented in the last section.

## 2. Materials and Methods

### 2.1. Study Area

The study area is Shenzhen (22°27′–22°52′ N, 113°46′–114°37′ E), a major city in Guangdong Province, China. Shenzhen is located within the Pearl River Delta, the southern of Guangdong Province, neighboring Hong Kong. Due to the policy of ‘reform and opening’, Shenzhen was promoted to the China First Special Economic Zone (SEZ) in the 1980s. Over 40 years, Shenzhen—once a tiny town—has grown into a modern metropolis. To improve urban management, the local government reorganizes the administrative structure for three levels: districts, sub-districts, and communities. Shenzhen has 10 districts which have been traditionally divided into guannei (e.g., Futian, Luohu, Yantian, and Nanshan), known as core distircts, and guanwai (i.e., Bao’An, Longgang, Longhua, Pingshan, Guangming, and Dapeng). With a permanent population of 11.9 million, the city covers an area of 1997.27 square kilometers. By the end of 2016, Shenzhen had the fourth-ranking GDP in China, following Shanghai, Beijing and Guangzhou, and a GDP per capita of 167,411 CNY, which was the top among Chinese cities [62].

Local governments have been pouring efforts into the economy and social benefits, as well as into green benefits. Shenzhen was the first city in China to win the international awards for livable communities (The LivCom Awards) in 2000. In a study of livable cities in China by the Chinese Academy of Sciences, Shenzhen was the most suitable city with respect to natural environment among 40 cities in 2016 [63]. Currently, the green coverage rate has reached 45.08% in Shenzhen’s built-up areas [64]. By the end of 2020, there will be more than one thousand parks in Shenzhen. According to the local government’s policies and regulations on urban green space planning, urban parks are subdivided into three levels: community/neighborhood parks, city parks, and natural (forest and country) parks. Community parks with small size were designed for meeting the demand of surrounding residents’ daily recreation, relaxation and physical exercise. City parks with acreage over 0.05 km^2^ have functional zones, amusement areas and recreation facilities for residents’ cultural and activities every week. Natural parks which are usually located at the suburbs, are full of forest resources or natural landscapes to prevent disordered urban construction and maintain ecosystem balance. They are good places for urban residents’ monthly outdoor activities. In this paper, all parks considered are free to enter for residents. There are 745 community parks, 117 city parks, and 23 natural parks. Table 1 presents descriptive statistics of parks in Shenzhen’s districts. At the city level, park area per capita is 0.58 for community parks, 4.26 for city parks, and 22.44 for natural parks, respectively. The value of park area per capita in Bao’an District, which has the largest population, is below the average level at three levels of park. The level of natural park area per capita in Yantian District and Dapeng District is far above the average. During the processing, we overlaid park polygons with districts polygons and found nine parks cross several districts. If any parts of park polygon was inside, the number of park in that district increased by 1. Thus, the total number of parks grouped by three park levels at the district level is equal or greater than that grouped by the three levels of park at city level. 

### 2.2. Data Source and Preprocessing

**Figure 1:**
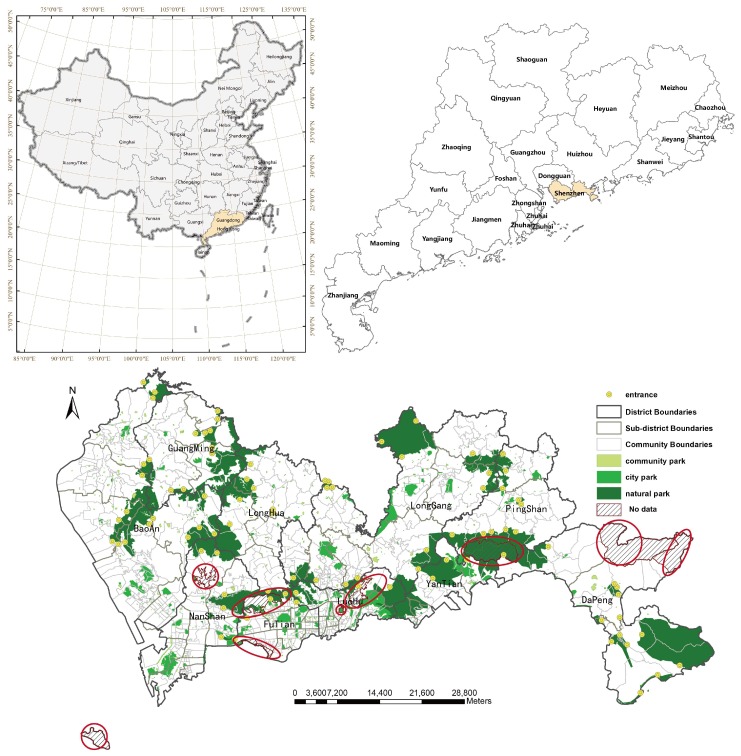
Study area and the distribution of parks in 2014.

Data were obtained from Urban Planning, Land and Resources Commission of Shenzhen Municipality, including the administrative boundaries, parks, and building population. Because the community is the smallest geographical unit of administrative management in Shenzhen and is the finest geographic resolution with demographics and socio-economic information, it was chosen as the basic analysis scale. Shenzhen encompassed 639 communities under jurisdiction of 57 sub-districts (Figure 1). As stated in Table 1, the polygon of parks were categorized into three levels: community parks, city parks, and natural parks. In total, 885 parks were chosen into this study. The parks vary in size, functions, amenities, and designed service radius. For the community parks and city parks, the geometric centroids were used as the service sites location. While the entrances (*n* = 90) of natural parks were digitalized from the document of Master Plans For Shenzhen Forest City (2016–2025) [65] and georeferenced to the China Geodetic Coordinate System 2000 (CGCS2000). Some may argue the entrances of community and city parks are better than geographic centroids. In fact, no reliable, open data sources are available to collect over hundreds of parks entrances for research. In some cases, any point along the perimeter of community parks can serve as the entry point [23,25]. Given these facts, the geometric centroids were chosen as the proxy for community parks and city parks.

The population-weighted centroids were calculated as Equations (1) and (2) to represent the demand of community population [66,67].

(1) $$x_{c}=\;\frac{\sum_{i}^{m_{c}}x_{i}*p_{i}}{\sum_{i}^{m}p_{i}}$$

(2) $$y_{c}=\;\frac{\sum_{i}^{m_{c}}y_{i}*p_{i}}{\sum_{i}^{m}p_{i}}$$

where *x_c_* and *y_c_* are the coordinates of population-weighted centroid of community *c*, *x_i_* and *y_i_* indicate the coordinates of the geometric centroid of the *ith* building within community *c*, *p_i_* denotes the population of the building, and *m_c_* is the total number of buildings within community *c*. After overlay and aggregation operation in ArcGIS 10.3 (Environmental Systems Research Institute, Inc., Redlands, CA, USA), there are nine communities without building’s geometric centroid located inside. We highlighted the locations of nine communities without population data by dark red polygons and slash patches. Two communities were located in Nanshan District, the north one was a reservoir, while the south one was an island. Two communities were in the middle of Luohu District. Two communities belonged to Futian District. The community located in Pingshan District is named Maluan. Two communities were located at the east corner of Dapeng District. Therefore, only 630 communities with population data were chosen as the demand sites and analysis units.

### 2.3. Method

#### 2.3.1. Modified Gaussian-Based 2SFCA

In this study, we followed the method proposed by Dai [27], a Gaussian-based 2SFCA method to calibrate the potential accessibility to urban parks in Shenzhen. The Gaussian-based 2SFCA method is an integration of 2SFCA and a Gaussian formed continuous decay function. The generalized 2SFCA model involves two steps:

Step 1: Generating the service catchment with a threshold travel cost for each park *j*, searching all populations within the catchment and computing the park area-to-population ratio *R_j_*, as

(3) $$R_{j}=\frac{S_{j}}{\sum_{i\in \left\{d_{ij}\;\leq \;d_{m}\right\}}P_{i}f\left(d_{ij}\right)}$$

Step 2: Generating the population demand catchment with a threshold travel cost for each population location *i*, searching all parks that are within the catchment, and summing up the park area-to-population ratios as

(4) $$A_{i}=\underset{k\in \{d_{ik\;}\leq \;d_{m}\}}{\sum }R_{k}f\left(d_{ik}\right)$$

where *R_j_* is the park-to-population ratio in park *j*, *S_j_* is the attractiveness of park *j*, *P_i_* denotes the population of community *i* within the catchment of park *j*, *d_ij_* is the travel cost between community *i* and park *j*, *f(d_ij_)* is the generalized cost decay function, *d_m_* presents the catchment radius. *A_i_* represents the park accessibility of population at location *i*.

As one of the most popular impedance functions [68], the Gaussian function discounts accessibility with a low rate of declination, instead of dividing subzones within a catchment such as in the E2SFCA method. In this study, the Gaussian function is formulated as

(5) $$G\left(d_{ij},\;d_{0},d_{m}\right)=\;\{\begin{array}{c} 1,\;if\;d_{ij}\;\leq d_{o}\\ \frac{\;e^{-\left(1/2\right)\times \frac{\left(d_{ij}-d_{o}\right)}{\left(d_{m}-d_{o}\right)}}-e^{-\left(1/2\right)}}{1-\;e^{-\left(1/2\right)}}\\ 0,\;\;\;if\;d_{ij}>\;d_{m}\;\; \end{array},\;if\;d_{o}<d_{ij}\leq \;d_{m}\;$$

In Equation (5), *d_ij_* is the travel cost between community *i* and park *j*, *d_o_* is the start point of accessibility discount, and *d_m_* is the threshold of travel cost. This Gaussian function, assumes that within a certain range people do not care about the travel cost, which means that the probability that people visit the parks equals 1. When the travel cost rises up to a certain limit, the probability is 0. If the cost *d_ij_* is between *d_o_* and *d_m_*, the probability of visiting park *j* would be between 0 and 1. After replacing *f(d_ij_)* in Equation (4) with Equation (5), the index *A_i_* is defined as

(6) $$A_{i}=\underset{k\in \{d_{ik\;}\leq d_{m}\}}{\sum }R_{k}G\left(d_{ik},d_{o},d_{m}\right)=\underset{k\in \{d_{ik\;}\leq d_{m}\}}{\sum }\frac{S_{k}G\left(d_{ik},d_{o},d_{m}\right)}{\sum_{j\in \{d_{jk\;}\leq d_{m}\}}P_{j}G\left(d_{jk},d_{o},d_{m}\right)}$$

#### 2.3.2. Estimation of Travel Distance

In this study, the travel distance is chosen to calculate the accessibility. Some scholars have argued that the travel time is the optimum measure of spatial impediment between demanders and facilities [69,70]. In reality, the speed of travel varies with transport mode. It is difficult to attain the actual speed of different transport modes in the entire city, instead of pre-defined speeds according to road classifications [35,60,71]. The estimated travel time will vary along with the defaulted speed of travel. While the travel distance is the length of the transport network from origins to destinations, it is fixed for a longer time than the travel time.

**Figure 2:**
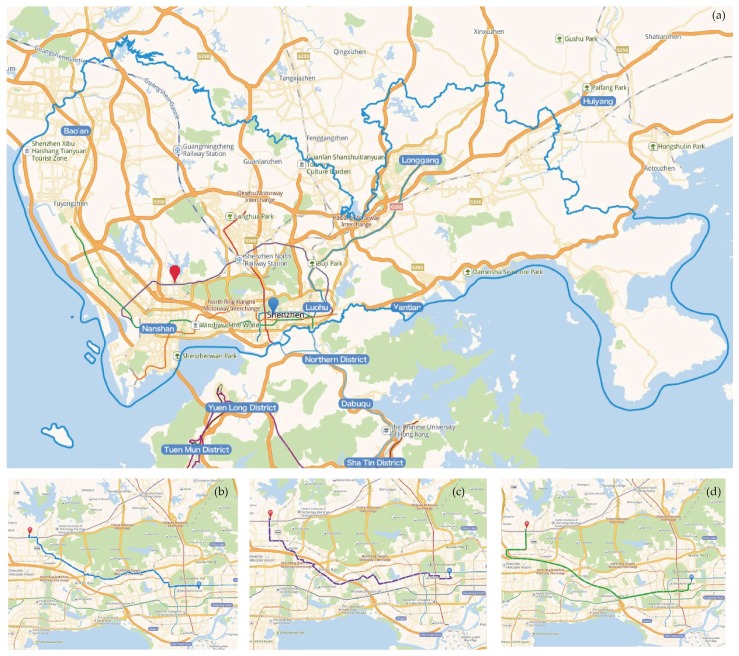
Screenshot of road network in Shenzhen and an example of four types of distance: (**a**) the Euclidian distance; (**b**) the walking distance; (**c**) the riding distance; (**d**) the driving distance. Datasource: Amap from AutoNavi Software Co., Ltd. (Beijing, China) Origin (blue marker) = (114.070173, 22.546626), Destination (red marker) = (113.948956, 22.582773).

To estimate the travel distance with diverse transport modes between 630 communities and 885 urban parks, we utilize the AutoNavi Maps API (http://lbs.amap.com/) from AutoNavi Software Co., Ltd., which is one of the leaders in providing web mapping, navigation, and location-based services in China. The service provider collected independent network datasets for walking, driving, bicycling and public transit. By spider crawling, we requested AutoNavi Maps API about 599,760 (630 × 952) times to obtained four types of distance for modelling in August 2018, including Euclidian distance, walking distance, bicycling distance, and driving distance (Figure 2). Particularly, 56,700 (630 × 90) calculation times were completed for the distance between natural entrances and communities.

#### 2.3.3. Parameters

Potential accessibility to parks is influenced by various factors, such as location, quality of park facilities, landscape, surrounding environment, and travel cost between the demand populations and park provision. In this study, a modified Gaussian-based 2SFCA method (MG2SFCA) is adopted to assess the accessibility to parks. The attractiveness *S_j_* of park *j* pertains to many factors, such as park size, landscape, number of amenities and park safety [33,41]. open-access data about parks’ characteristics throughout the entire city are unavailable. Moreover, the location and size are usually fixed, while other characteristics can be changed easily [48]. Larger parks which offer more amenities can attract residents from further away have are positively associated with higher rates of park access [41]. Many guidelines and regulations in urban planning defined open space standards related to the size of open space [72]. For example, the standards under The Act on Urban Parks, Greenbelt, etc. is 6 m^2^ per person. Compared to the subjective attractiveness from park quality, which is usually based on questionnaire or field survey [38], size is unbiased [38]. Thus, the size of parks is used as the attractiveness. In the MG2SFCA model, the parameters *d_m_* and *d_o_* are crucial because they influence the catchment size and decay rate. The choice of catchment size *d_m_* usually varies among authors. For park service catchment size, the standards from acts and regulations pertaining to urban parks are indicative references for researchers [34,37,73]. In Shenzhen, the local government has set a green goal for 2020: citizens can access a community park within 0.5 km, a city park within 2 km, and a natural park within 5 km. According to the document of Outline of Urban Greening Planning in Shenzhen 2012–2020, all citizens could access a community park within 2 km, a city park within 5 km, and a natural park within 10 km in 2015. For population catchment size, some scholars have argued that people in metropolitan or rural setting can vary [57,58,59]. After readjusting administrative divisions for several times, the extent of Shenzhen Special Economic Zones has covered the entire city since 2010. In this study, the catchment sizes of park service and population are set to the same value. The parameter *d_o_* is based on the assumption that within a certain range of travel costs, the probability that people visit parks is always 1. However, empirical data on the distance at which people’s interest in visiting parks do not decrease are scarce. We adopted the standards from the Outline of Urban Greening Planning in Shenzhen. The low standards in 2015 were adopted as the parameter *d_m_*, and the 2020 goal as the parameter *d_o_*. Details about *d_o_* and *d_m_* for the three levels of park were shown in Table 1.

#### 2.3.4. Measurement of Spatial Disparity

In essence, the metric of MG2SFCA is still the ratio of service-to-demand, similarly to 2SFCA. In this case, the value is the average park area per capita, and the population-weighted MG2SFCA of all communities is equal to the ratio of total park area to total population. In a community, the higher Ai is, the better park accessibility becomes. If a high accessibility community has a high population density, the underlying utilization of park services is likely to be higher. If residents of a low population density area are provided with high park accessibility, there will be a surplus of park service. To determine the areas with a shortage of park services using the degree of supply-to-demand match, accessibility and population density in communities are standardized as

(7) $$z_{A_{i}}=\frac{A_{i}-\bar{A}}{\sigma_{A}}$$

(8) $$z_{PopD_{i}}=\frac{PopD_{i}-\bar{PopD}}{\sigma_{PopD}}$$

where *Z_Ai_* is the Z-score of *A_i_* and $\bar{A}$ denotes the ratio of total park area to total population. *PopD_i_* is the population density of community *i*, *Z_PopDi_* is the Z-score of *PopD_i_*, $\bar{PopD}$ is the population density in the study area, and *δ_PopD_* is the standard deviation of the population density. The combination of *Z_Ai_* and *Z_PopDi_* can be utilized to calibrate the match degree between park provision and demand population in community *i*:

(1) If *Z_Ai_* > 0 and *Z_PopDi_* > 0, community *i* has high accessibility and high population density.
(2) If *Z_Ai_* < 0 and *Z_PopDi_* < 0, community *i* has low accessibility and low population density.
(3) If *Z_Ai_* > 0 and *Z_PopDi_* < 0, community *i* has high accessibility and low population density.
(4) If *Z_Ai_* < 0 and *Z_PopDi_* > 0, community *i* has low accessibility and high population density.

## 3. Results

### 3.1. Proximity between Communities and Urban Parks under Four Types of Distance

#### 3.1.1. Proximity without Distance Threshold

Travel distance is an effective measure of proximity between origins and destinations. Table 2 shows variations in proximity among four distance types. Statistically, the mean value of all routes for Euclidean distance was 23.52 km, followed by the values for walking (mean = 29.96 km), bicycling (mean = 31.74 km), and driving (mean = 32.02 km). The Euclidean distance was the shortest, and the driving distance was the longest. The difference between the Euclidean distance and driving distance is significant, i.e., a difference of 8.5 km. There were no significant differences between bicycling and driving distance.

In order to estimate the degree correlation among the four distance types, a global correlation analysis was performed. From the correlation matrix in Table 3, the four types of distance were strongly correlated with each other, with high correlation coefficient values over 0.95. Second, Euclidean distance was most strongly correlated with the other three types of distance (>0.98), but the correlations of driving distance with other types of distance were the weakest. The correlation between walking distance and bicycling distance was nearly 1. Thus, if it is impossible to obtain the bicycling network dataset in the whole city, the walking network can be a valid alternative.

#### 3.1.2. Proximity under Fixed Distance Threshold

**Figure 3:**
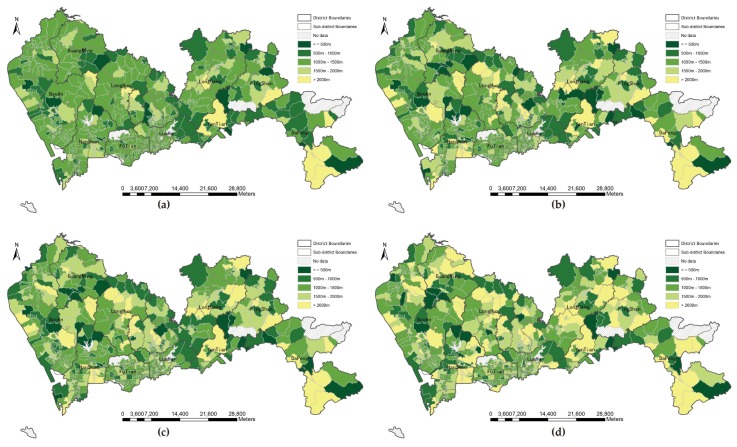
Proximity to community parks in threshold of 2 km by four types of distance: (**a**) Euclidean distance; (**b**) walking distance; (**c**) bicycling distance; (**d**) driving distance.

**Figure 4:**
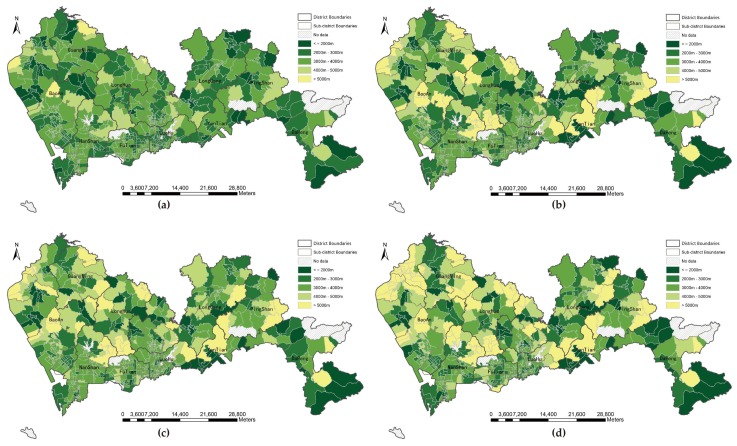
Proximity to city parks in threshold of 5 km by four types of distance: (**a**) Euclidean distance; (**b**) walking distance; (**c**) bicycling distance; (**d**) driving distance.

**Figure 5:**
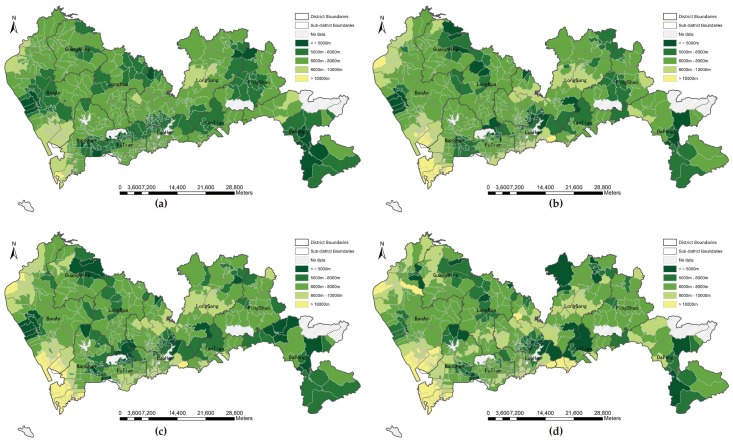
Proximity to natural parks in threshold of 10 km by four types of distance: (**a**) Euclidean distance; (**b**) walking distance; (**c**) bicycling distance; (**d**) driving distance.

Although the proximity results without tolerance thresholds indicated high correlations among the four types of distance, the local variations at three levels of parks are still unknown. Taking the service threshold of different level parks into account, we summarized four types of mean travel distance within catchment thresholds for three levels of parks and divided the mean distance into five groups, as shown in Figure 3 (community parks, 2 km), Figure 4 (city parks, 5 km), Figure 5 (natural parks, 10 km). As T the mean travel distance increases, the color gradually changes to yellow. A community filled with yellow indicated that no parks are available within the catchment threshold. For three levels of parks, Figure 3a, Figure 4a, and Figure 5a are greener than the other three distance modes. Euclidean distance returns a larger number of communities within the distance threshold than walking distance, bicycling distance, F and driving distance do. Interestingly, in according with the global correlation analysis, the patterns of walking distance and bicycling distance are similar with each other among the three levels, e.g., Figure 3b,c, Figure 4b,c, and Figure 5b,c. If a bicycle network is unavailable, the walk network can be a suitable substitute. The average shortest network distance by car within the threshold was the longest, and the number of yellow communities was greater than that for other types of distance. Therefore, the metric of distance is vital to the assessment of potential accessibility.

### 3.2. Results of MG2SFCA at Three Levels of Parks

#### 3.2.1. Statistical Analysis of Park Accessibility

As stated in Section 3.1.2, the threshold for catchment size was 2 km for community parks, 5 km for city parks, and 10 km for natural parks. Table 4 shows the statistical accessibilities for three levels of parks under four types of distance. The results suggest that (1) In general, the average and standard deviation of park accessibility at natural level are much higher than those at the community level and city level. This finding may be related to the large area of natural parks in Shenzhen. (2) Regardless of the metric of travel distance used, the accessibility at the natural park level is better than that at the other two levels of parks. The number of underserved communities, the area of underserved communities, and the underserved population are lowest. (3) With respect to transportation mode, the number of underserved communities, the area of unserved communities and the unserved population are the lowest for Euclidean distance, but the largest for driving distance. This finding indicates that the park accessibility assessed by MG2SFCAwithin a fixed catchment size is easy to overestimate by the metric of Euclidean distance. People who opt to drive to parks may travel farther than those who walk or cycle.

#### 3.2.2. Spatial Analysis of Park Accessibility

**Figure 6:**
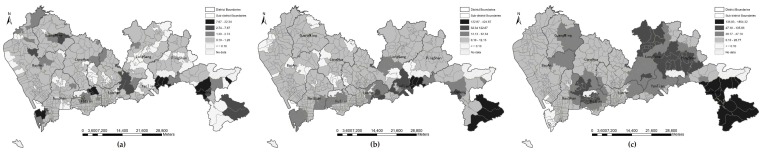
Park accessibility by Euclidean distance at three levels: (**a**) community park level; (**b**) city park level; (**c**) natural park level.

**Figure 7:**
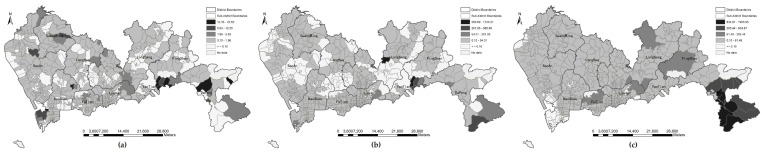
Park accessibility by walking distance at three park levels: (**a**) community park level; (**b**) city park level; (**c**) natural park level.

**Figure 8:**
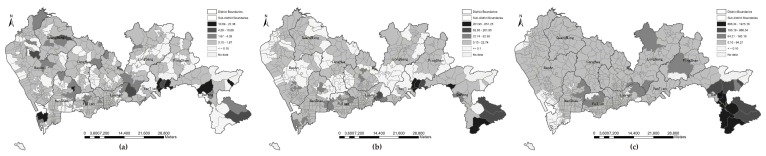
Park accessibility by bicycling distance at three park levels: (**a**) community park level; (**b**) city park level; (**c**) natural park level.

**Figure 9:**
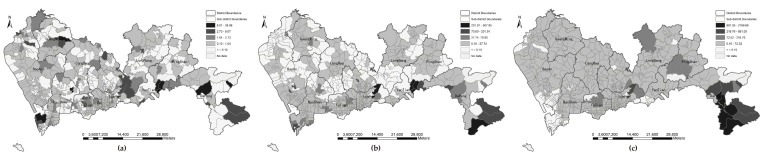
Park accessibility by driving distance at three park levels: (**a**) community park level; (**b**) city park level; (**c**) natural park level.

The statistical differences in park accessibility under the four metrics of travel distance were analyzed as indicated above, but the spatial distribution and patterns of park accessibility are still unclear. Figure 6, Figure 7, Figure 8 and Figure 9 show the accessibility of the 639 communities to urban parks for Euclidean distance, walking distance, bicycling distance, and driving distance, respectively. Dark color indicates high accessibility. In particular, the white denotes that a community is underserved; that is, the accessibility value is less than 0.1. At first glance, the park accessibility at three levels under any metric of travel distance is better in the central city, such as in Futian and Luohu Districts. because the reason is that the number of underserved communities is less than that of other districts. By comparison with Figure 6, the accessibility measured by Euclidean distance is better than that measured by the other types of travel distance (e.g., walking distance (Figure 7), bicycling distance (Figure 8), and driving distance (Figure 9). This finding reminds us that the metric of distance deserves attention when calculating accessibility byMG2SFCA. The number of darker communities is greater, while the white communities is lower. The spatial patterns of accessibility in Figure 7, Figure 8 and Figure 9 are similar. A comparison in Figure 7, Figure 8 and Figure 9 shows that regardless of the metric of travel distance is used, apparent variations in spatial accessibility exist among the three level of parks. For instance, in Figure 7 (walking distance), the spatial distribution of underserved communities at the community park level is discrete in throughout the whole city, while at the city park level the underserved communities cluster together. For natural parks, most of the underserved communities are at edge of the administrative territory. Generally, the spatial accessibility at natural park level is higher than that at the community and city levels, i.e., Figure 7c is greyer than Figure 7a,b. Notably, the darkest communities at the natural park level is at the south of Dapeng, while the communities are white at the community level.

### 3.3. Disparity Analysis of the Supply-to-Demand

**Figure 10:**
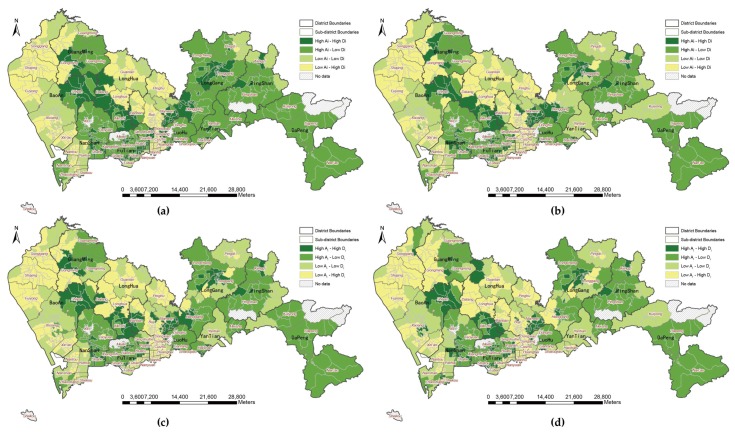
Park accessibility and its associations with population density under four types of travel distance: (**a**) Euclidean distance; (**b**) walking distance; (**c**) bicycling distance; (**d**) driving distance.

The ratio of park acreage to population at the three levels of park is 0.58 for community parks, 4.26 for city parks, and 22.44 for natural parks, respectively. Generally, the distribution of natural parks has a significant effect on the total park accessibility such that most communities with high accessibility are surrounded by natural parks. It should be noted that the size of natural parks are larger than most of city parks and communities parks. Meanwhile, the accessibility values positively correlated with park area and negatively with population. Taking all parks into calculation, the accessibility at the natural park level accounts for over 80% of the total. By summing the park accessibility at three levels, the total accessibility (Ai) values are standardized by Equation (7) to conduct a joint analysis with the standardized population density by Equation (8). The spatial patterns of the association between total park accessibility and population density is shown in Figure 10. Under four types of distance, communities with high accessibility but low population density are mainly distributed in the western and southern sub-districts, including Pingshan, Dapeng, and Nan’ao. However, communities with low accessibility and high population density, which show a severe mismatch between park provision and demand population, are located in Songgang, Shajing, Fuyong, Xixiang, and Xin’an in Bao’an District; Gongming in Guangming District; Guanlan and Longhua in Longhua District; and Pinghu, Bantian, and Buji in Longgang District. Migrant workers live in those deprived communities where an abundance of factories have opened and low living costs are needed.

From a statistical perspective (Table 5), the variation of the associations between total park accessibility and population density under different types of transportation distance excluding Euclidean distance is relatively unobvious with respect to the number of communities. Some 18% of the communities have high population density and high park accessibility, while less than 17% of the communities are low-density areas with low park accessibility. That is, over half of the communities have an accessibility level inappropriate for their population density. The percentage of communities that have high population density but low park accessibility is over 40% (equivalent to approximately 55% of the population).

## 4. Conclusions

This study introduced an innovative model, named as modified Gaussian-based 2SFCA (MG2SFCA), to explore the disparity between park provision and the demand population in terms of potential spatial accessibility. Unlike previous studies that used a dichotomous function or a continuous decay function in 2SFCA or Gaussian-based 2SFCA model [27,52], a constant and gradual mixed impedance function was adopted to delineate the interactions between park provision and the demander, and differentiate spatial variation within catchment areas. The modified Gaussian function was based on rational assumption that within a limited amount of travel distance, visitors’ willingness to go to a park is not diminished. In a defaulted range of distance, the possibility decrease between 1 and 0. Additionally, the result of MG2SFCA is the ratio of park acreage to population, a intuitive reflection of the availability of green space resources [74]. As one member of the 2SFCA family, this method can be easily extended to evaluate accessibility of facilities such as health care, food outlets, and amenities.

Distance is a key parameter to calibrate the spatial separation between the supply and the demand in spatial accessibility modeling. In this paper, we constructively explore the influence of the metric of travel distance on spatial accessibility. In a case of Shenzhen, China, four types of travel distance between communities and urban parks was requested by online map API service. By contrast and correlation analysis among four types of distance (e.g., Euclidean distance, walking distance, bicycling distance, and driving distance), the correlations were shown to be relatively high between alternative types of distance, which are aligned with prior study [75]. Under the method of MG2SFCA, the accessibility calculated by Euclidean distance was overestimated relative to that calculated by the other three types of distance. Notably, the patterns of proximity and accessibility in Shenzhen under walking and bicycling distance were similar. It implied that a network dataset by walking is an alternative to a detailed, unavailable network dataset that includes bike lanes and cycle tracks. This study should encourage urban planners to consider the metric of distance on the evaluation of green spaces provision and environmental justice from perspective of spatial accessibility.

Taking urban parks’ function, size, and location into account, we evaluated the disparity between park provision and the demanders in terms of accessibility at hierarchical levels (e.g., community park, city park, and natural park) under four types of distance. The statistical and spatial variation of park accessibility at three levels is apparent. In general, at the natural park level, accessibility is better, and the number of underserved communities and the underserved population is the smallest. The underserved communities are distributed at the edge of the administrative boundary. At the community park level, the distribution of underserved communities is scattered throughout the whole city, but clustered at the city park level. The disparity analysis showed that the mismatch between park provision and demand population is better in core districts (Luohu, Futian, Nanshan, and Yantian) than that in non-core districts [76]. Communities, which suffer from a severe shortage of park service provision due to the high population density as well as the low spatial accessibility, were located in Songgang, Shajing, Fuyong, Xixiang, and Xin’an in Bao’an District; Gongming in Guangming District; Guanlan and Longhua in Longhua District; and Pinghu, Bantian, and Buji in Longgang District. The findings can give some implications for urban planners and policy-makers on delineating shortage areas and where to locate additional parks to meet residents’ fundamental physical and psychical demands from visiting urban green spaces.

Several limitations are worth addressing and of improvement. First, although the population data at the building level is available, a population-weighted centroid was used as a proxy for the demand population in a community. It was based on the assumption that all populations within the community have uniform park accessibility. Actually, the number of buildings in Shenzhen was over 0.6 million. It is time-consuming to harvest the four types of distance of building-to- park in the whole city by AutoNavi Maps API for individual users. Second, the catchment size was the same for both the supply and demand side by consulting urban planning regulations and acts. It remained unidirectional without reference to park visitors’ preference. Third, only urban parks and population in Shenzhen are considered to assess accessibility. Without available data in neighboring cities, this study cannot account for the edge effect of park accessibility. Although the distance between origins and destinations was considered, we neglected the influence of routes which people go to parks through on the park accessibility [49]. In the future, we will attempt to eliminate aforementioned limitations to offer more practical evaluation of park accessibility.

## Figures and Tables

**Table 1:** Descriptive statistics of parks in Shenzhen’s districts.

District	Population (Million)	Number of Park			Total Park Area (km^2^)			Park Area per Capita (m^2^)		
		Community Park	City Park	Natural Park	Community Park	City Park	Natural Park	Community Park	City Park	Natural Park
Futian	0.76	147	12	2	0.60	7.24	7.35	0.79	9.57	9.72
Luohu	0.92	133	10	3	0.66	10.85	18.22	0.72	11.74	19.72
Yantian	0.17	32	8	2	0.27	5.86	44.67	1.61	35.13	267.97
Nanshan	1.17	65	28	1	0.94	12.23	9.91	0.80	10.45	8.47
Bao’an	4.56	142	15	4	2.71	6.30	34.51	0.59	1.38	7.56
Longgang	3.45	103	18	7	1.35	15.16	56.13	0.39	4.40	16.28
Longhua	2.29	46	5	5	0.86	0.87	43.44	0.37	0.38	18.97
Pingshan	0.46	28	6	3	0.14	2.22	38.14	0.31	4.84	83.31
Guangming	1.06	28	4	4	0.62	1.31	28.80	0.58	1.23	27.14
Dapeng	0.10	21	12	3	0.46	1.57	54.11	4.40	15.00	517.84

Note: park area per capita is the ratio of total park area to population.

**Table 2:** Univariate statistics for the distances calculated between communities and parks.

Distance Type (km)	Mean	P10	Q1	Q2	Q3	P90
Euclidean distance	23.52	6.30	12.57	21.63	32.31	42.65
Walking distance	29.96	8.17	16.52	27.44	40.61	54.01
Bicycling distance	31.74	8.85	17.69	29.19	42.85	57.12
Driving distance	32.02	9.1	17.37	29.39	43.82	57.57

Note: P10 10th percentile, Q1 lower quartile, Q2 median, Q3 upper quartile, P90 90th percentile.

**Table 3:** Global Pearson correlations between alternative types of distance.

Distance	Euclidean	Walking	Bicycling	Driving
Distances between communities and parks (N)	599,760	599,760	599,760	599,760
Euclidean distance	1	0.986 **	0.985 **	0.981 **
Walking distance	0.986 **	1	0.997 **	0.973 **
Bicycling distance	0.985 **	0.997 **	1	0.972 **
Driving distance	0.981 **	0.973 **	0.972 **	1

Note: ** indicates significance at the 1% level.

**Table 4:** Statistical comparison of the different levels of park accessibility under four types of travel distance.

Level of Parks	Travel Distance Threshold	Distance Mode	Mean	Standard Deviation	Underserved Community (Numbers)	Underserved Areas (km^2^)	Underserved Population
Community park	2 km	Euclidean	0.82	1.85	79	328.38	2,336,132
		Walking	0.79	1.90	126	471.72	3,723,610
		Bicycling	0.78	1.89	137	541.78	4,125,854
		Driving	0.77	1.98	181	675.48	5,487,025
City park	5 km	Euclidean	9.98	28.90	35	165.08	1,515,039
		Walking	13.28	79.68	97	545.06	3,620,555
		Bicycling	11.70	49.19	100	566.75	3,766,218
		Driving	11.17	44.51	148	716.66	5,395,964
Natural park	10 km	Euclidean	54.82	211.38	5	24.59	77,714
		Walking	52.47	214.58	45	66.23	781,082
		Bicycling	52.27	215.24	59	88.92	1,092,732
		Driving	54.34	225.48	74	121.67	1,288,487

Note: The underserved is defined as the score of accessibility lower than 0.1 calculated byMG2SFCA.

**Table 5:** Summary of the associations between park accessibility (Ai) and population density (PopDi).

	Type of Distance	High A_i_—High PopD_i_	High A_i_—Low PopD_i_	Low A_i_—Low PopD_i_	Low A_i_—High PopD_i_
Count	Euclidean	151 23.97%	158 25.08%	71 11.27%	250 39.68%
	Walking	114 18.10%	138 21.90%	91 14.44%	287 45.56%
	Bicycling	113 17.94%	136 21.59%	93 14.76%	288 45.71%
	Driving	114 18.10%	125 19.84%	104 16.51%	287 45.56%
Population	Euclidean	4,246,057 28.42%	2,014,124 13.48%	1,380,564 9.24%	7,300,112 48.86%
	Walking	3,383,533 22.65%	1,520,657 10.18%	1,874,031 12.54%	8,162,636 54.63%
	Bicycling	3,435,872 23.00%	1,699,450 11.37%	1,695,238 11.35%	8,110,297 54.28%
	Driving	3,038,667 20.34%	1,512,910 10.13%	1,881,778 12.59%	8,507,502 56.94%

Note: The total number of communities = 630, excluding 9 communities without population data; the total population in the study area = 14.94 million.
